# Amended Ferrozine Assay for Quantifying Magnetosome Iron Content in Magnetotactic Bacteria.

**DOI:** 10.1021/acsomega.4c08607

**Published:** 2024-12-12

**Authors:** Ya-Chun Zhao, Li-Fen Wu, Siang Chen Wu

**Affiliations:** Department of Environmental Engineering, National Chung Hsing University, 145 Xingda Road, Taichung 40227, Taiwan

## Abstract

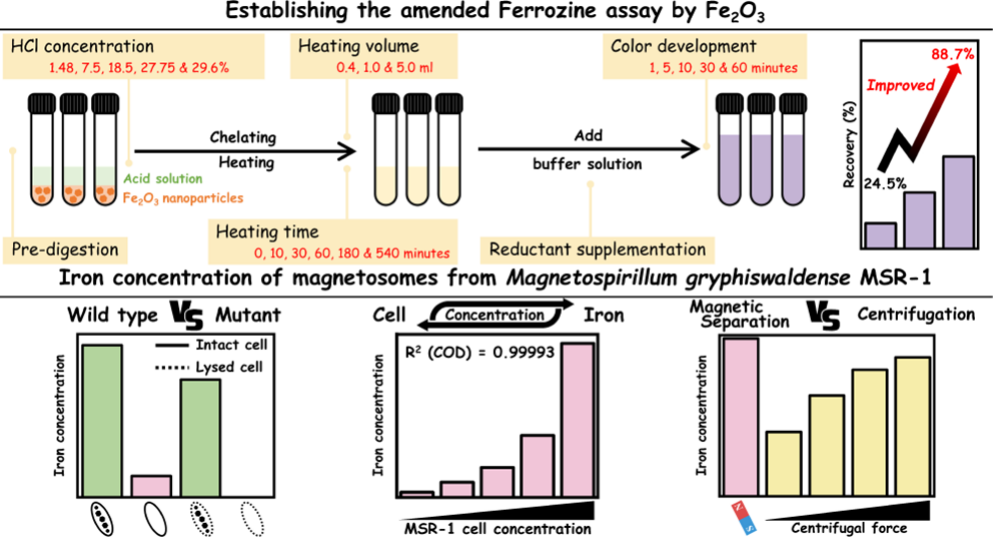

*Magnetospirillum gryphiswaldense* MSR-1 can biomineralize the magnetosome, nanoscale magnetite (Fe_3_O_4_) surrounded by a lipid bilayer, inside the cell. The magnetosome chain(s) enables MSR-1 to move along with the magnetic field (magnetoaerotaxis). Due to its unique characteristics, MSR-1 has attracted attention for biotechnological applications. During cultivation, not only the optical density but also the magnetosome content in MSR-1 should be monitored. The ferrozine assay had been utilized to quantify the iron content in magnetosomes. However, the effectiveness of the ferrozine assay on iron oxide nanoparticles is still unknown. Here, we examined the experimental factors, and the amended ferrozine assay demonstrates a recovery of 88.71% for Fe_2_O_3_ nanoparticles relative to the stock solution. Next, we apply the assay to analyze MSR-1 samples, which successfully reveals the difference in iron contents between magnetic and nonmagnetic MSR-1 samples and highlights the amount of MSR-1 cell density suitable for amended ferrozine assay. The assay further helps us examine the effects of centrifugation compared to magnetic separation (MS). The detection of residual magnetosomes in the supernatant indicates that MS remains a suitable method for collecting magnetosomes. We anticipate the amended ferrozine assay will facilitate research on MSR-1 by enabling investigators to measure iron content in cells in a fast, easy, and cost-effective manner.

## 1 Introduction

The *Magnetospirillum gryphiswaldense* strain MSR-1, a member of magnetotactic bacteria (MTB), can biomineralize Fe_3_O_4_ nanoparticles within cells known as magnetosomes when the pO_2_ is below 20 mbar.^1^ The magnetosome chain(s) acts as an internal compass, enabling MSR-1 to move in response to the magnetic field (magnetoaerotaxis).^2^ Due to their unique characteristics, both MSR-1 and magnetosomes have garnered significant attention for their potential applications in biomedical and biotechnological fields, such as hyperthermia treatment, enzyme immobilization, and wastewater treatment.^3^ However, nonmagnetic MSR-1 can be observed during cultivation, which can be attributed to high oxygen concentrations, a lack of iron sources, or the loss of genes responsible for magnetosome synthesis.^4,5^ To maintain MSR-1 cells in the lab, it is important to monitor not only the optical density (OD) but also the magnetosome content in the culture.

Common methods used for quantifying magnetosomes include transmission electron microscopy (TEM),^6^ magnetic response (*C*_mag_),^7^ inductively coupled plasma optical emission spectroscopy (ICP-OES),^8^ atomic absorption spectroscopy (AAS),^9^ and the ferrozine assay.^10^ TEM, coupled with ImageJ analysis, allows for direct counting and size analysis of magnetosomes in cells.^6^ To measure magnetic response, which is taking advantage of the magnetosome chain, MSR-1 cells are aligned vertically and horizontally with the light beam of a spectrophotometer, which results in different value of light scattering, and the measured values are then used to calculate *C*_mag_.^7^ ICP-OES, AAS, and the ferrozine assay are employed to measure the iron concentration in collected cell samples.^8−10^ However, apart from the ferrozine assay, the aforementioned methods require laborious sample preparation, expensive instruments, and maintenance and often necessitate the installation of additional apparatus. For example, *C*_mag_ requires investigators to install a customized electromagnetic system around the sample holder in the spectrophotometer.^11^ Furthermore, even though the strong permanent magnet could be an alternative of the customized electromagnetic system, the magnet may interrupt the measurement by obstructing part of the light path in the spectrophotometer. In contrast, the ferrozine assay utilizes a compound called ferrozine, which chelates with ferrous ions and forms a stable magenta solution. The magenta color can be easily detected using a spectrophotometer at a wavelength of 562 nm.^12^ Due to its simplicity and rapidity, we have chosen the ferrozine assay as the preferred method for quantifying magnetosome content in MSR-1.

The ferrozine assay has been widely used to assess the iron content in various samples. Initially, Viollier et al.^13^ applied the ferrozine assay to measure total iron concentration in potable water and later evaluated the concentrations of ferric iron and ferrous iron separately by executing the chelation and reduction reactions. Fish^14^ proposed an improved ferrozine method for quantifying total iron in biological samples. This method involved the use of potassium permanganate to release protein-complexed iron, which was then chelated by ferrozine after reduction. The improved ferrozine assay was subsequently adapted in studies that investigated iron oxide nanoparticle uptake in cancer cells.^15^ In addition to the biological samples, Braunschweig et al.^16^ utilized the ferrozine assay to analyze iron concentration in minerals, such as magnetite, goethite, and ferrihydrite. Quantification of iron concentration in MTB cells or magnetosomes was performed using the ferrozine assay based on the method described in the Viollier’s study.^4,10,17^ Briefly, the collected MSR-1 cells or magnetosomes were digested with 65% HNO_3_ for 3 h first and then analyzed by a modified ferrozine method. Iron particle digestion and soluble iron species analysis were separated. Furthermore, it should be noted that since the ferrozine assay was originally developed for soluble iron species, caution is necessary when dealing with solid iron nanoparticles, and the accuracy of the assay for such particles remains to be explored. The workflows and reagents employed in each of the aforementioned studies were varied, making it difficult to establish a standardized procedure. For instance, differences were observed in (1) the duration of heating (digestion) in Stookey’s study (10 min), Norouzi et al.’s study (1 h), and Braunschweig et al.’s study (24 h); (2) the volume of heating (digestion) in study of Riemer et al.^18^ (0.3 mL) and Braunschweig et al.’s study (1.0 mL), with no information provided in Stookey’s study; (3) the concentrations of hydrochloric acid used, with Fish’s study employing 0.6 N and Braunschweig et al.’s study using either 1 or 6 M; (4) the duration required for color development, specified as 15 min in Fish’s study but with no information provided in Norouzi et al.’s study; (5) the separation of chelation and reduction steps, observed in Norouzi et al.’s study but not in Riemer et al.’s study; and (6) the predigestion of samples, conducted in Braunschweig et al.’s and Norouzi et al.’s studies but not in Stookey’s study.

In this study, we assessed various experimental factors using Fe_2_O_3_ nanoparticles to evaluate the accuracy of the ferrozine assay for the nanoparticles. Based on the results, we established an amended version of the ferrozine assay that combined the digestion of iron nanoparticles and reduction of Fe^3+^ in one step. Furthermore, we utilized the amended ferrozine assay to analyze magnetosomes and MSR-1 cell samples. This not only demonstrated the applicability of the assay but also revealed the limitations of cell density of the MSR-1 sample used in the amended ferrozine assay. Lastly, we applied the amended ferrozine assay to examine different methods for magnetosome collection in MSR-1 samples under various growth conditions. Specifically, we compared centrifugation and magnetic separation, and the results showed that magnetic separation remains the most suitable method for collecting magnetosomes.

## 2 Methods

### 2.1 Reagents

The water used in the study was high purity water with a resistivity of 18.2 MΩ·cm (Merck Millipore), unless otherwise indicated. All glassware were soaked in a 1.2 M HCl solution overnight and then rinsed with deionized water. The acid solution consisted of 0.01 M ferrozine, 1.44 M NH_2_OH·HCl, and 6.0 M HCl in water. The buffer solution contained 5.12 M CH_3_CO_2_NH_4_ and 2.7 M concentrated NH_4_OH in water. The standard iron solution (125 Fe^2+^-mg/L) was prepared by dissolving 0.002 M FeSO_4_·7H_2_O and 0.06 M HCl in water. For the Fe_2_O_3_ suspension (125 Fe^3+^-mg/L), 1.8 mg of Fe_2_O_3_ nanoparticles with a particle size of less than 50 nm (Sigma-Aldrich) were resuspended in 10.0 mL of water. The Fe_2_O_3_ nanoparticles were used as substitutes for magnetosomes in the ferrozine assay for different factors. The NH_2_OH·HCl solution contained 1.44 M NH_2_OH·HCl in water. The inorganic washing buffer (IWB) consisted of 1.0 mL of EDTA chelated trace element mixture,^19^ 7 × 10^–4^ M KH_2_PO_4_, and 0.004 M NaNO_3_ in deionized water (pH = 7.0). IWB without the trace mineral mixture was designated as IWB-M.

### 2.2 Amended Ferrozine Assay

Before the analysis, the acid solution was heated on a heating plate at 80 °C with stirring to prevent solute precipitation. The sample was placed in a test tube, and acid solution was added. The resulting mixture was then heated at 100 °C. After the mixture cooled to room temperature, the buffer solution was added, and the solution was transferred to a 10.0 mL volumetric flask. It was then diluted to the mark with water and transferred to a clean test tube. The development of the magenta color was allowed to fully occur. The absorbance was measured at 562 nm. Additionally, the pH of the solution was measured to confirm that it fell within the range of 4.0 to 9.0, ensuring maximum and stable absorbance.

To understand the recovery efficiency of the ferrozine assay for quantifying iron nanoparticles, the effects of heating time, heating volume, reductant supplementation, color development time, hydrochloric acid concentration, and predigestion procedure were evaluated. For the evaluation of heating time, 0.2 mL of Fe_2_O_3_ suspension was mixed with 0.2 mL of the acid solution and 0.6 mL of water to make a heating volume of 1.0 mL. The mixture was heated at 100 °C for 10, 30, 60, 180, and 540 min. To assess the effect of the heating volume, 0.2 mL of Fe_2_O_3_ suspension was initially mixed with 0.2 mL of the acid solution. Then, 0.0, 0.6, and 4.6 mL of water were added to the mixture to achieve heating volume of 0.4, 1.0, and 5.0 mL, respectively. The effects of the reductant were evaluated by providing an additional 0.2 mL of the 1.44 M NH_2_OH·HCl solution to the heated samples. The effects of color development time were assessed by measuring the absorbance and pH of samples after the addition of the buffer solution for 1, 5, 10, 30, and 60 min. To examine the effects of hydrochloric acid concentration, acid solutions with varying concentrations (0.48, 2.4, 6.0, 9.0, and 9.6 M) were prepared for the ferrozine assay. The effects of the predigestion procedure were investigated by mixing 1.0 mg of Fe_2_O_3_ nanoparticles with different predigestion solutions in a test tube, including 6.0 M HCl solution (hereafter referred to as 6.0 M), 6.0 M HCl solution with 0.01 M ferrozine (hereafter referred to as 6.0 MF) or 1.44 M NH_2_OH·HCl (hereafter referred to as 6.0 MN), and acid solution. The mixed solution was heated at 100 °C for 10 min as the initial digestion of the nanoparticles, followed by dilution to 10.0 mL in a volumetric flask. For analysis, 0.2 mL of the predigested Fe_2_O_3_ solutions were used as samples, mixed with 0.2 mL of the acid solution, and then analyzed.

### 2.3 Strain and Bacterial Growth Conditions

*Magnetospirillum gryphiswaldense* MSR-1 (DSM 6361) was obtained from Deutsche Sammlung von Mikroorganismen and Zellkulturen (DSMZ, Brunswick, Germany) and stored in a 20% glycerol stock at −20 °C. A mutant strain of MSR-1, B17316, which lost the magnetotaxis trait, was occasionally obtained in the laboratory.^20^*M. gryphiswaldense* MSR-1 cells and B17316 were cultivated in flask standard medium (FSM)^1^ in a 30 °C incubator at 120 rpm agitation. MSR-1 was regularly grown in 5.0 mL of FSM in a 10.0 mL serum bottle with a butyl rubber stopper with 1% O_2_ and 99% N_2_ in the headspace. The MSR-1 cells were subcultured every 3 days. For preculture of MSR-1, 0.5 mL of the regular MSR-1 cell culture was first inoculated into 5.0 mL FSM and cultivated microaerobically for 48 h. Next, 5.0 mL of MSR-1 cell culture was inoculated into 35.0 mL of FSM with in a 100.0 mL serum bottle, followed by microaerobic cultivation for 16 h. Subsequently, MSR-1 cultures were collected and washed twice in IWB at 8500 rpm for 10 min at 4 °C. The washed MSR-1 cells were then introduced into a 200.0 mL serum bottle containing 100.0 mL of FSM. The bottle was sealed with a butyl rubber stopper, and the headspace was maintained at atmospheric gas composition to enable MSR-1 oxygen consumption during cultivation and create conditions conducive to magnetosome biomineralization. The initial OD_565_ of MSR-1 cells was controlled at 0.05. The MSR-1 cells were subsequently incubated in a 30 °C incubator with agitation at 120 rpm for 24 h. For cell sample collection, the MSR-1 cultures were first pelleted at 8,500 rpm for 10 min at 4 °C and then washed twice in IWB-M. The OD_565_ of the cell culture was adjusted to 2.0, and then 1.0 mL of the cell culture was dispensed into Eppendorf tubes. MSR-1 cells were pelleted again at 8,500 rpm for 10 min at 4 °C by centrifugation. The supernatants were decanted, and the cell pellets were stored at −80 °C. Unless otherwise indicated, the cell density of MSR-1 used in the magnetosome purification test was OD_565_ 2.0.

### 2.4 Magnetosome Purification

For obtaining crude cell extracts (CCE), the collected cell pellets were lyzed by resuspending them in a 2.0 M KOH solution and heated at 80 °C for an hour.^21^ After being cooled to room temperature, the CCE was transferred to an Eppendorf tube. The tubes were placed on a magnetic rack (catalog no. 1005, Sergi Lab Supplies) overnight. The supernatant was carefully decanted by pipetting, and the magnetosomes were washed twice in 10× PBS and three times in water. To resuspend the magnetosomes, they were mixed with 10× PBS and magnetically separated overnight. The supernatant was decanted the next day, and the resuspend–separate–decant step was repeated. The magnetosomes were then resuspended in water, and the resuspend–separate–decant step was repeated three times. Finally, the magnetosomes were resuspended in 0.2 mL of water and stored at 4 °C until analyzed by the amended ferrozine assay.

### 2.5 Application of Amended Ferrozine Assay on Magnetosomes

**Figure 1:**
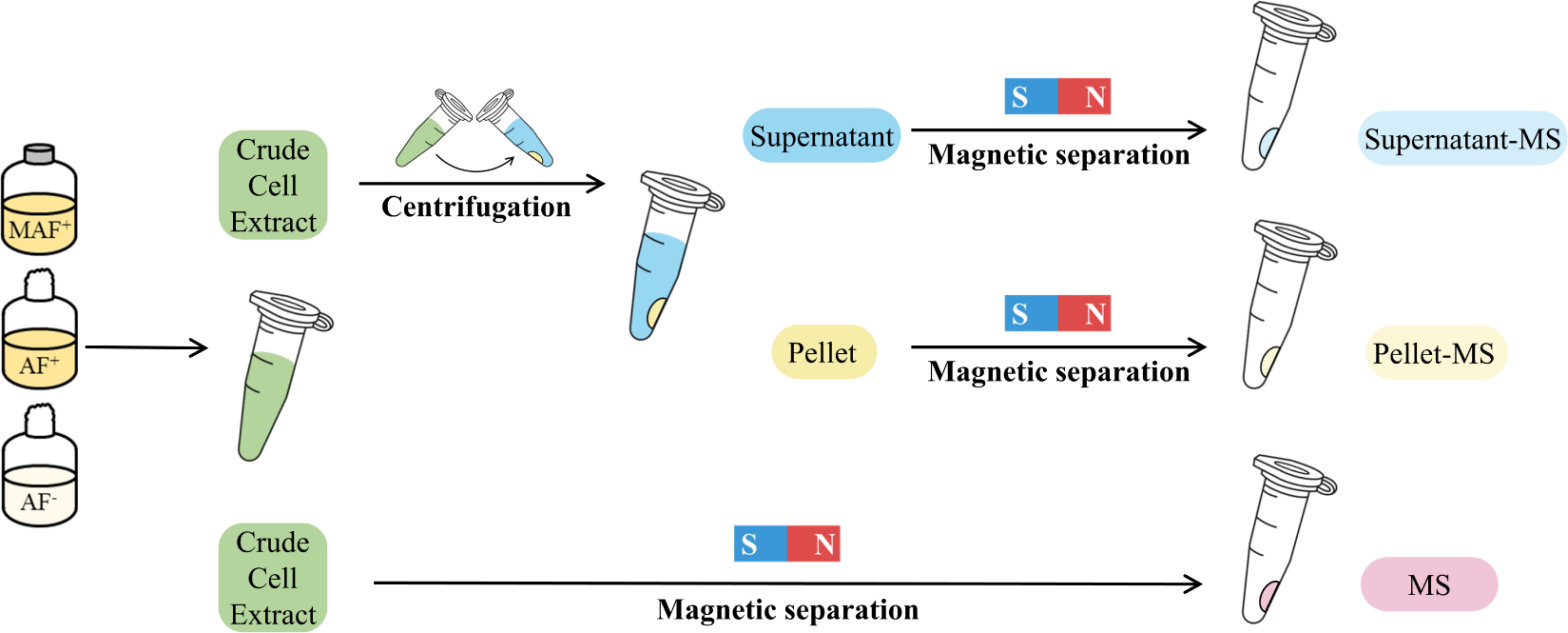
Schematic diagram of evaluating the different magnetosome-collected methods on MSR-1 samples from different growth conditions (MAF^+^, AF^+^, and AF^–^).

To assess the iron concentration in wild-type MSR-1, CCE was obtained using the aforementioned methods. Magnetosomes in the CCE of wild-type MSR-1 cells were collected magnetically. The iron concentration of CCE and magnetosomes were analyzed by an amended ferrozine assay. Sample with a volume of 0.2 mL was mixed with 0.2 mL of acid solution (6.0 M HCl). After heating at 100 °C for 10 min, the buffer solution was added, and the sample volume was adjusted to 10.0 mL in a volumetric flask. The absorbance was measured at 562 nm. To evaluate the iron concentration with different cell density at OD_565_, the MSR-1 cells were first pelleted by centrifugation at 8500 rpm for 10 min at 4 °C and then washed twice in IWB-M. The OD_565_ of the cell culture was adjusted to 0.2, 0.5, 1.0, 2.0, and 5.0 by dilution with IWB-M. Next, 1.0 mL of each OD_565_ cell culture was dispensed into Eppendorf tubes, and the MSR-1 cells were pelleted by centrifugation. The supernatants were removed by pipetting, and the cell pellets were lysed as previously mentioned above. Magnetosomes in each OD_565_ from the cell culture were collected by magnetic separation (MS) and then analyzed using the amended ferrozine assay. To evaluate the different magnetosome collection methods (as depicted in Figure 1), we prepared the CCE of wild-type MSR-1 cells under various growth conditions. MSR-1 cells cultivated as described above, with an atmospheric gas composition in the headspace and 100 μM ferric citrate in the FSM medium, were denoted as MAF^+^. In the final step of the preculture procedure, MSR-1 cells cultivated in a serum bottle covered with a cotton plug instead of a butyl rubber stopper were designated as AF^+^ indicating aerobic cultivation with 100 μM ferric citrate in the FSM medium. The AF^–^ indicated that MSR-1 was cultivated aerobically, and the iron source in FSM was omitted. The magnetosomes in CCE were either collected by centrifuging at 206, 825, 6624, and 16,708 × *g* for 10 min at 4 °C or by magnetic separation. The supernatant (S) and pellet (P) of each centrifugation speed was collected, and the pellet was resuspended in 0.2 mL of water. The magnetosomes in the supernatant and cell pellet that were further collected by magnetic separation were denoted as S-MS and P-MS, respectively. The iron concentration of the collected samples was analyzed using the amended ferrozine assay.

### 2.6 Analytical Method

To benchmark the amended ferrozine assay against the standard method, we simultaneously conducted several experimental conditions simultaneously. These conditions included (condition 1): A heating volume of 5.0 mL and a heating time of 10 min; (condition 2): A heating volume of 0.4 mL and a heating time of 10 min; and (condition 3): A heating volume of 0.4 mL and a heating time of 10 min with supplementation of NH_2_OH·HCl solution. In conditions 1 and 2, the sample used was a Fe_2_O_3_ nanoparticle suspension, whereas in condition 3, a standard iron solution was employed. Briefly, the samples were digested as previously described. After cooling to room temperature, we either diluted the samples to 15.0 mL and filtered them through a 0.22 μm filter or analyzed them using the amended ferrozine assay. The iron content in former samples was analyzed using inductively coupled plasma optical emission spectrometry (ICP-OES, Thermo Scientific). All of the experimental conditions were performed in triplicate.

## 3 Results and Discussion

### 3.1 Establishing the Amended Ferrozine Assay by Fe_2_O_3_

**Figure 2:**
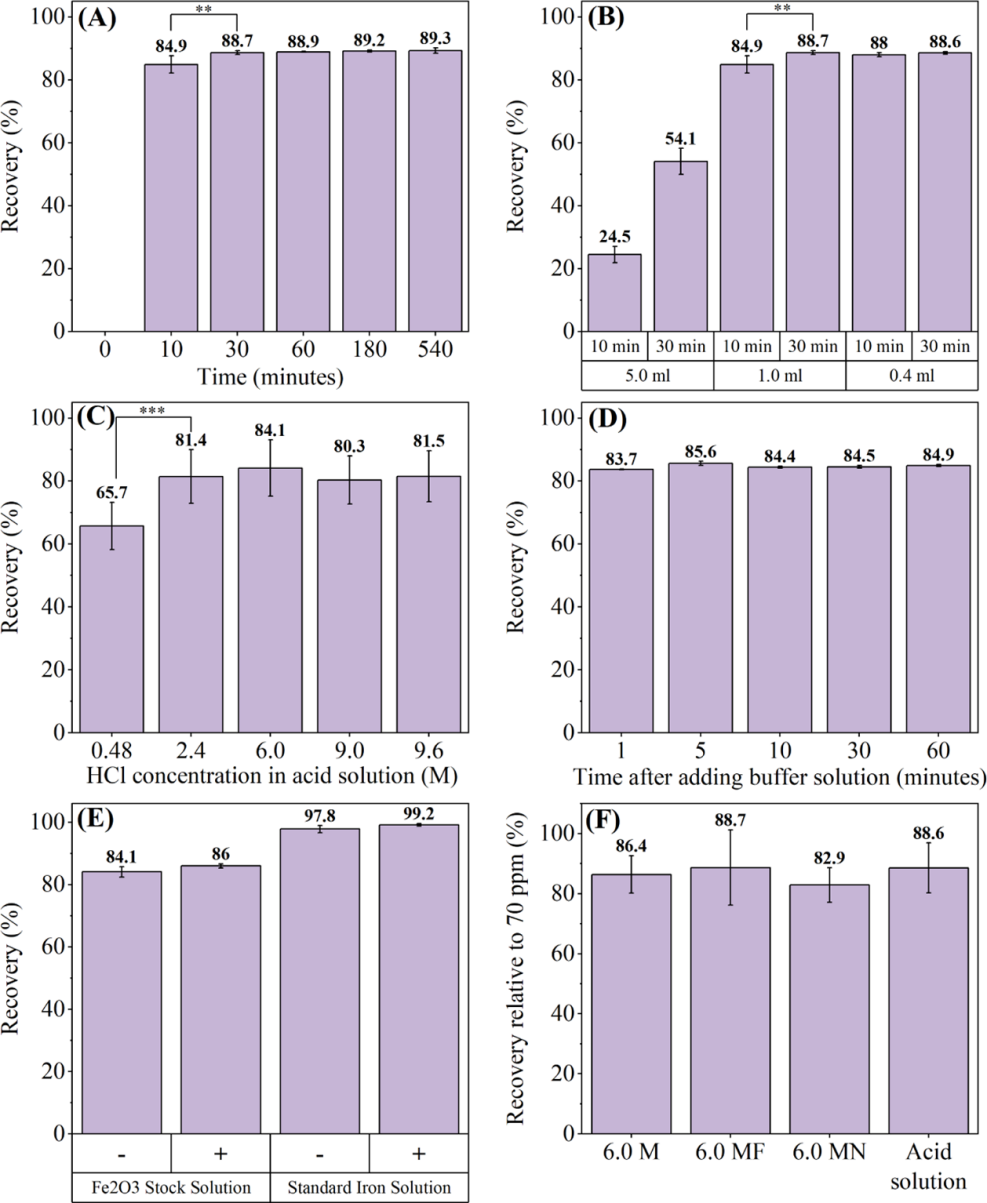
Evaluating of different experimental factors in the ferrozine assay. (A) The effects of heating time. (B) The effects of heating volume. (C) The effects of HCl concentration in acid solution. (D) The time for the development of color. (E) The effects of the reductant (+: With supplementation of reductant solution; −: Without supplementation of reductant solution). (F) The effects of the predigestion procedure (M: molarity; MF: 6.0 M HCl solution with 0.01 M ferrozine; MN: 6.0 M HCl solution with 1.44 M NH_2_OH·HCl). Asterisks indicate statistically significant differences in recovery between samples (*: *p* < 0.05; **: *p* < 0.01; ***: *p* < 0.001).

To establish the ferrozine assay for analyzing the iron concentration of magnetosomes, we first assessed the limit of detection of the ferrozine assay. Using soluble iron which ranged from 0 to 10 ppm, the iron concentration of 5 ppm showed a decrease in the OD_562_ value (0.209 ± 0.004) comparing to 2.5 ppm (0.293 ± 0.005) (Data not shown). The decrease in value could be attributed to the decrease in pH, which leads to inefficient color development in the samples. Based on the results, we next assessed the effects of different heating times on recovery, which was calculated against the known concentration stock solution (2.5 ppm) (Figure 2A). The recovery was 84.86% when the solid Fe_2_O_3_ nanoparticles were heated for 10 min. Extending the heating time to 540 min, the recovery of the Fe_2_O_3_ nanoparticles reached 89.32%, which was the highest recovery. We observed a significant difference between the recoveries at 10 and 30 min (88.71%), indicating that increasing the heating time improves the recovery of Fe_2_O_3_ nanoparticles within the first 30 min. However, there was no significant difference in recovery between 30, 60, 180, and 540 min (*p* > 0.9), suggesting that 30 min is sufficient to achieve the recovery when the heating volume is 1.0 mL.

Next, we tested the effects of different heating volumes on the recovery (Figure 2B). With a heating volume of 5.0 mL, the recovery at 10 and 30 min was 24.52% and 54.08%, respectively. When the heating volume was limited to 1.0 mL, the recovery at 10 and 30 min increased to 84.86% and 88.71%, respectively. However, reducing the heating volume to 0.4 mL resulted in a recovery of 87.96% at 10 min and 88.56% at 30 min, with no significant difference in recovery (*p* > 0.99). These results indicate that the recovery of Fe_2_O_3_ nanoparticles increases with decreasing heating volume and further imply that with increasing acid concentration. Based on these findings, the heating time can be reduced to 10 min while controlling the heating volume at 0.4 mL. We further assessed other factors with a heating volume of 0.4 mL and a heating time of 10 min.

The original concentration of HCl in the acid solution was 6.0 M,^12^ and we further explored the effects of HCl concentrations (0.48, 2.4, 9.0, and 9.6 M in the acid solution) (Figure 2C). The lowest recovery was observed with 0.48 M HCl in the acid solution. Although the 6.0 M HCl concentration showed the highest recovery, the differences in recovery between 2.4, 6.0, 9.0, and 9.6 M HCl concentrations were not significant (*p* > 0.64). These results indicate that a 0.48 M HCl concentration is insufficient for digesting the Fe_2_O_3_ nanoparticles and increasing the HCl concentration improves recovery, albeit reaching a plateau. The 6.0 M HCl concentration in the acidic solution was chosen for the following experiment.

We tested the time required for developing the magenta color within the samples in this study (Figure 2D). The samples were left at room temperature for 1, 5, 10, 30, and 60 min(s) after adding the buffer solution, and the absorbance at 562 nm was measured at each time point. The recovery reached 83.72% at 1 min and 85.64% at 5 min, indicating that 5 min is sufficient for developing the magenta color in this study. Extending the time beyond 5 min did not further affect recovery. To assess the effects of the reductant, an additional NH_2_OH·HCl solution was added to the heated samples. Both the standard iron solution and the Fe_2_O_3_ nanoparticle solution were tested as samples (Figure 2E). Based on the results, the recovery with and without the NH_2_OH·HCl solution supplement for the Fe_2_O_3_ samples was not significantly different (*p* = 0.4), indicating that the reductant supplement did not affect the recovery.

Here, we adjusted a few experimental factors and tested the effects of predigestion of the solid Fe_2_O_3_ nanoparticles. A 1.0 mg sample of Fe_2_O_3_ was first digested by different predigestion solutions, including 6.0 M HCl, 6.0 MF (6.0 M HCl solution with 0.01 M ferrozine), and 6.0 MN (6.0 M HCl solution with 1.44 M NH_2_OH·HCl), and an acid solution, without any additional transfer of nanoparticles. After diluting to 10.0 mL, the digested solutions were used as iron stock solutions (70 Fe^3+^-mg/L) for the ferrozine assay. The results (Figure 2F) showed that the recovery in all four groups was similar, ranging from 82.93% to 88.7% (*p* >0.35). These results indicate that the predigestion of nanoparticles did not affect recovery. Furthermore, while developing the amended ferrozine assay, we have assayed the effect of predigestion of Fe_2_O_3_ nanoparticles with concentrated nitric acid. The nitric-digested Fe_2_O_3_ solution was used as samples and analyzed by a ferrozine assay, in which the iron concentration was 2.085 ± 0.013 ppm. Compared to the nitric-undigested Fe_2_O_3_ solution that was directly analyzed by the ferrozine assay (2.035 ± 0.012 ppm), the difference in iron concentration is not significant.

To compare the amended ferrozine assay with the standard method, we evaluated the recovery between the two methods (Figure S1). In condition 1, where Fe_2_O_3_ nanoparticles were digested in 5.0 mL for 10 min, the amended ferrozine assay showed a higher recovery than ICP-OES. However, there were no significant differences in recovery between the two methods in condition 2, where Fe_2_O_3_ nanoparticles were digested in 0.4 mL for 10 min. In condition 3, where the standard iron solution was digested in 0.4 mL for 10 min with the subsequent addition of a reductant, ICP-OES demonstrated higher recovery compared to the amended ferrozine assay. The recovery trends between ICP-OES and the amended ferrozine assay remained consistent in each condition, particularly showing no significant difference in condition 2. This suggests that the amended ferrozine assay can serve as an alternative method for measuring iron concentration in iron nanoparticles when the digestion procedure involves 0.4 mL heating volume and 10 min of heating time.

In this section, we examined factors including heating time, heating volume, HCl concentration in acid concentration, color development time, the supplement of reductant, and predigestion procedure. Among the tested factors, heating time, heating volume, and HCl concentration affected the recovery, while the other factors did not. Based on these results, the heating time of 10 min and the 6.0 M HCl concentration in the acid solution were retained from the original procedure, and the heating volume was controlled at 0.4 mL, which showed similar recovery with ICP-OES.

The ferrozine assay was initially utilized to analyze soluble iron in the water samples and was later extended to include solid iron oxide nanoparticles. However, no studies have assessed the applicability and accuracy of the ferrozine assay for iron oxide nanoparticles compared with standard nanoparticle suspensions. Additionally, the workflow and reagents of the ferrozine assay have been found to be varied and cumbersome for investigators. In this study, we examined the experimental factors based on the study of Stookey,^12^ and we retained the same reagents and workflow. The results presented in Figure 2 demonstrate that the heating volume, heating time, and HCl concentration have an impact on recovery, while other factors do not. High heating volume (5.0 mL) and low HCl concentration (0.48 M) significantly decrease recovery when heated for 10 min, which can be attributed to the reduced opportunity for iron nanoparticles to react with HCl. In both scenarios, recovery can be improved by increasing the heating time or HCl concentration in the acid solution. In the study by Tokar et al.,^22^ by incubating the iron oxide powders with continuous mixing for 7 days in acid solution with varied concentration, it was also observed that the dissolution efficiency of iron oxide increased with increasing HCl concentration. i.e., hematite reached 99.0% at 5.0 M HCl concentration, and magnetite reached 100% at 2.5 M HCl concentration. Based on the findings of our study, we retained the 6.0 M HCl concentration in the acid solution and a heating time of 10 min from the original study, and the highest recovery was achieved by controlling the heating volume to 0.4 mL. This recovery under the amended procedure was further compared with that under ICP-OES, and no significant difference was observed. These results suggest that the amended ferrozine assay could serve as a cost-effective alternative to ICP-OES. In the studies by Braunschweig et al.;^16^ Norouzi et al.;^15^ and Viollier et al.,^13^ where the heating step was avoided and NH_2_OH·HCl was added after digestion, we speculated the NH_2_OH·HCl could lose the ability as reductant when heated. However, in Figure 2E, the results showed that the addition of NH_2_OH·HCl after heating did not affect the recovery. Furthermore, a study by Cisneros et al.^23^ revealed that hydroxylamine hydrochloride/water (35 mass %) started to decompose at 145 ± 10 °C, indicating that the NH_2_OH·HCl may not decompose during the heating step in our study. In summary, the NH_2_OH·HCl did not affect recovery in the ferrozine assay, specifically due to decomposition of the NH_2_OH·HCl. There was no need to execute digestion and reduction separately.

### 3.2 Iron Concentration of Magnetosomes

**Figure 3:**
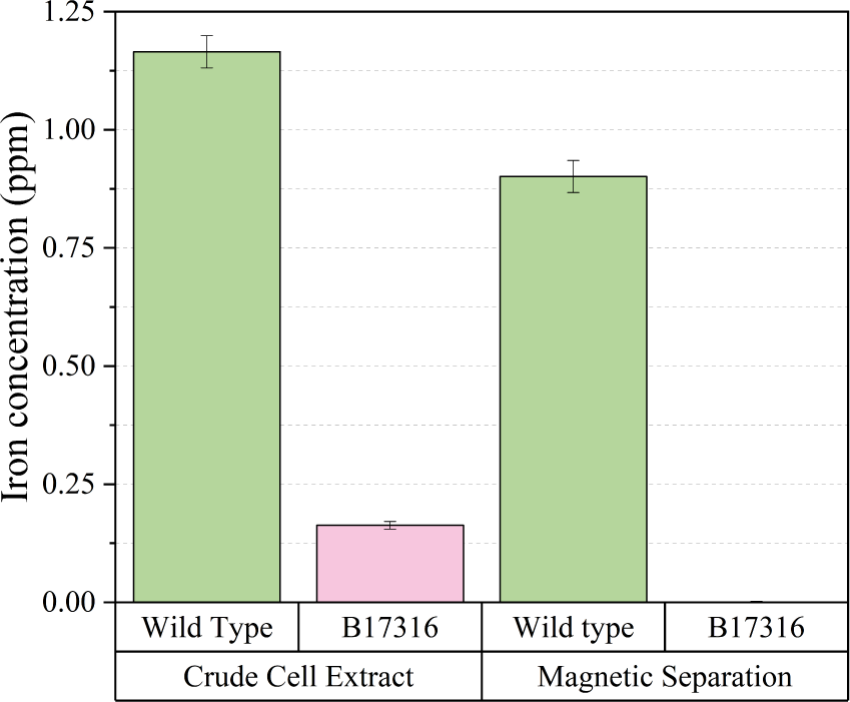
Application of amended ferrozine assay to wild-type (magnetic) and B17316 (nonmagnetic) MSR-1 cells.

To test the applicability of the amended ferrozine assay to magnetosomes, we initially applied the method to analyze the CCE and magnetosomes of wild-type and mutant strains (B17361) of MSR-1 cells. The B17361 is an magnetoaerotaxis-deficient strain which show 99.92% 16s rRNA gene similarity to MSR-1 wild type^20^ but lose few of the core magnetosome island (MAI) gene, including the essential magnetosome genes *mamO*(24) or *mamM*.^25^ (Figure S2 and Table S1) As depicted in Figure 3, the iron concentration of magnetic separation in strain B17361 was 0 ppm. Based on these results, we deduced that the measured iron concentration of the CCE in strain B17361 represented the inherent intracellular iron concentration (0.163 ppm). Conversely, the iron concentration of magnetic separation in wild-type MSR-1 was 0.9 ppm, which is attributable to the presence of magnetosomes. The difference in iron concentration between the CCE and magnetosomes in wild-type MSR-1 was 0.264 ppm, which can be attributed to the removal of inherent intracellular iron during the magnetic separation process. The difference in CCE between wild-type MSR-1 (0.264 ppm) and strain B17361 (0.163 ppm) could be attributed to the mutation of iron-transport-related genes that requires further examination in the future. According to the result, magnetosome concentrations take ∼77% of the iron concentration in CCE, which is higher than the results reported in two studies that showed 45% and 50% of iron content related to magnetosomes.^21,26^ However, when cultivating the MSR-1 cell in a well-controlled oxystat fermentor, the magnetosome concentrations increase to 99.5% of the total iron content of the cell.^27^

This result demonstrates that the amended ferrozine assay can distinguish between magnetic (wild-type) and nonmagnetic (B17361) MSR-1 cells.

**Figure 4:**
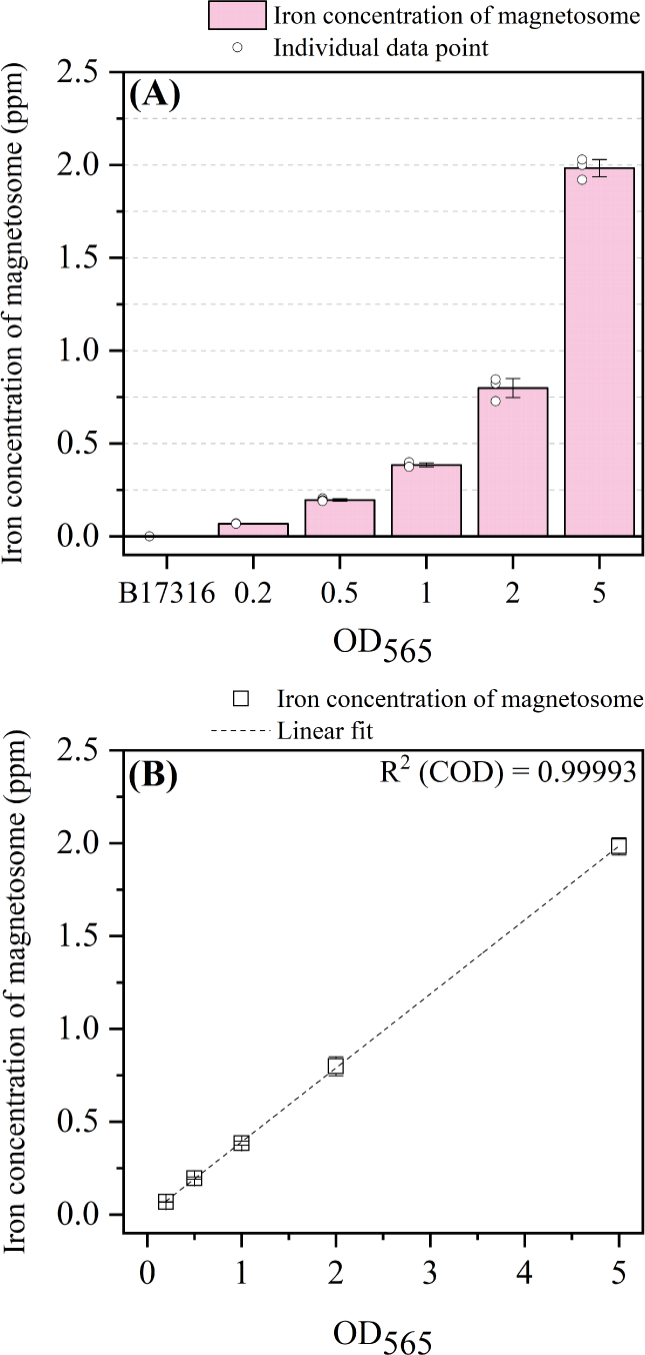
(A) Iron concentration of magnetosomes from variable OD_565_ of MSR-1. (B) Linear relationship between iron concentration and OD_565_.

Next, to determine the limitations of the MSR-1 cell density in the amended ferrozine assay, we prepared different OD_565_ samples from wild-type MSR-1 cells (Figure 4A). The iron concentration of magnetosomes in OD_565_ 0.2 samples was 0.068 ppm, while in OD_565_ 5.0 samples, it was 1.983 ppm. Furthermore, a linear relationship between the iron concentration and OD_565_ was established (Figure 4B). In our previous results, OD_565_ values of 0.01, 0.05, 0.1, and 10 were also assessed. For OD_565_ less than 0.2 and as diluted as 0.01, the iron concentration was negative due to the calculation of OD_562_ based on the linear regression of the calibration curve. The iron concentration of OD_565_ equal to 10 is 2.933 ppm, which did not show a fold change compared to 5 (2.076 ppm). (Figure S3) These results confirm the suitable range of OD_565_ for the MSR-1 samples, which falls between 0.2 and 5.0. Cell samples with an OD_565_ outside this range should be either concentrated or diluted before being analyzed by using the amended ferrozine assay. The iron concentrations in different OD_565_ samples were also determined in nonmagnetic strain B17361 with MSR-1 wild type as positive control (Figure S4). The 0 ppm was observed in all magnetic separation samples, which is the same as that in Figure 3.

**Figure 5:**
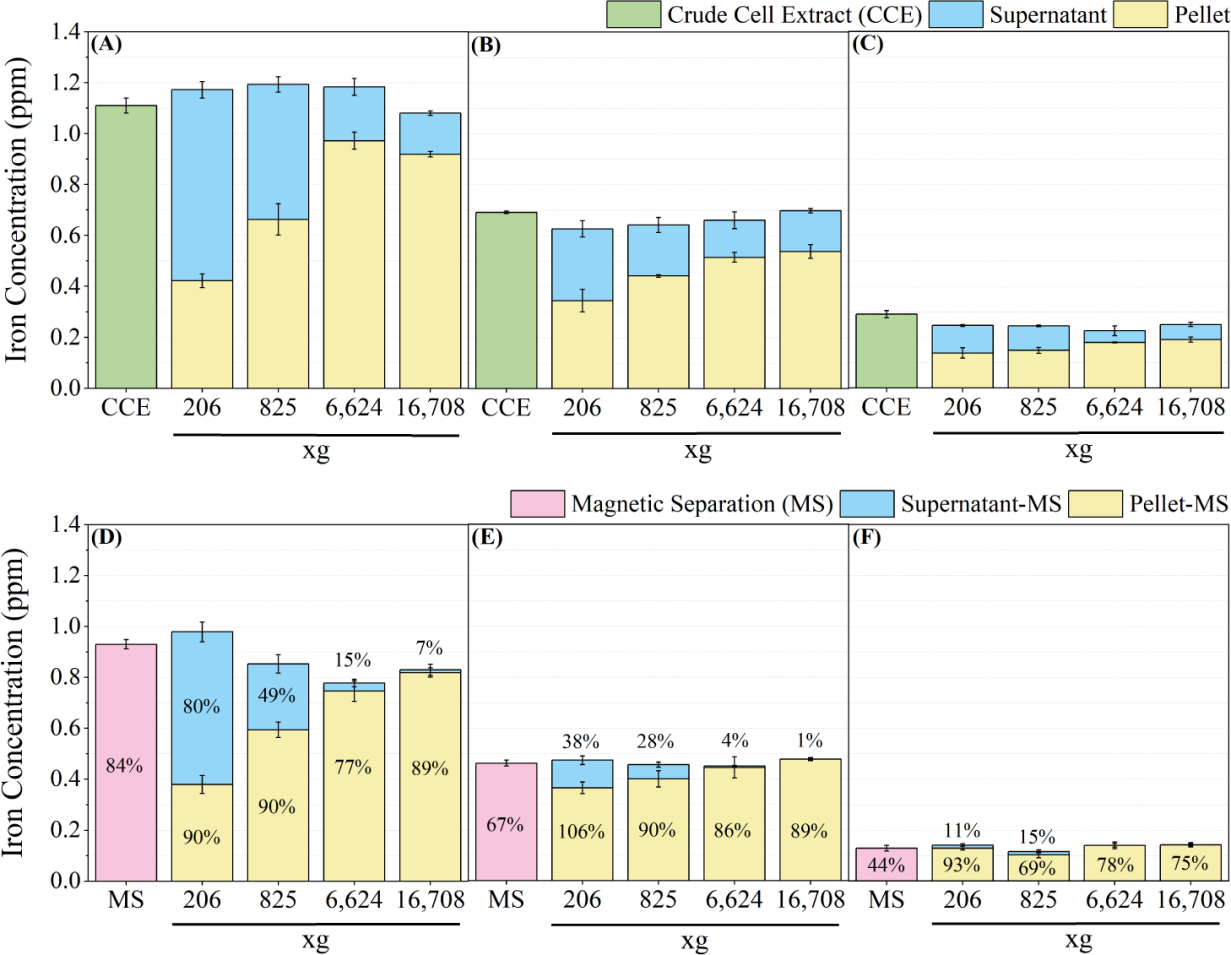
Comparison of magnetosome collection methods between centrifugation and magnetic separation on MSR-1 samples from different growth conditions, including MAF^+^ (A and D), AF^+^ (B and E), and AF^–^ (C and F). The number in the bar represents the percentage of the iron concentration of the sample after magnetic separation compared to that before magnetic separation. i.e., in (D), the iron concentration of magnetic separation takes 84% in crude cell extract.

In order to wash and collect magnetosomes, the entire process of our magnetic separation procedure takes 5 days, while centrifugation can be completed in less than an hour. The time-saving nature of centrifugation is undoubtedly attractive. Here, we collected magnetosomes from CCE by centrifuging the samples at speeds of 206, 825, 6624, and 16,708 × *g* or by using the MS method. The supernatants and cell pellets obtained from centrifugation were subsequently subjected to magnetic separation to examine the presence of residual magnetosomes. The iron concentrations of the collected samples were quantified by using the amended ferrozine assay. The samples were categorized based on cultivation conditions, centrifugal speed, sample type, and whether they were treated with MS or not. For example, in Figure 5A, the iron concentration of the pellet obtained from centrifugation at 206 × *g* was labeled as MAF^+^-206-P. In Figure 5D, the iron concentration of the pellet from 206 × *g* that underwent MS treatment was labeled as MAF^+^-206-P-MS. The value labeled in Figure 5D–F is calculated by dividing the iron concentration after and before magnetic separation in a given sample. For a value larger than 100% which could contribute to the experimental operation during magnetosome purification, the magnetosomes could lose when the supernatant is removed. Although the percentage of pellet of 206 × *g* pellet in Figure 5E is higher than in Figure 5B, the difference in iron concentration is not significant with a *p*-value of 0.87. In Figure 5A, even though the total iron concentrations of 206, 825, and 6624 × *g* are higher than CCE, the total iron concentration is not significant. The slight difference in iron concentration could also be attribute to the experimental operation. The results presented in Figure 5 demonstrate that the iron concentration in the pellet increases with an increasing centrifugal speed. The difference in iron concentration between MAF^+^-6624-P and MAF^+^-16,708-P is not significant (*p* = 0.42). The iron concentration in the magnetic separation treated pellet takes 69% to 106% compared to that in the magnetic separation untreated pellet samples. The results could be attributed to residual supernatant or the collection of cell debris. In contrast to the cell pellet, the iron concentration in the supernatant decreases with an increasing centrifugal speed. Figure 5E,F shows that the iron concentration of AF^–^-6624-S-MS and AF^–^-16,708-S-MS was 0 ppm, indicating the absence of residual magnetosomes in the supernatant. However, magnetosomes are still present in MAF^+^-6624-S-MS (15%) and MAF^+^-16,708-S-MS (7%) (Figure 5D). Moreover, the difference between MAF^+^-MS and MAF^+^-16,708-P-MS is significant (Figure S5), indicating that centrifugation at 16,708 × *g* for 10 min is insufficient to fully collect magnetosomes from the CCE despite its time-saving characteristic. Magnetic separation remains the appropriate method for magnetosome collection.

We employed the amended ferrozine assay to analyze MSR-1 crude cell extracts and magnetosomes, successfully distinguishing between magnetic and nonmagnetic MSR-1 samples. Our results indicate that the suitable range of cell density in the amended ferrozine assay lies within an OD_565_ range from 0.2 to 5.0. The upper and lower limits of cell density are determined by the calibration curve. For OD_565_ less than 0.2, the iron concentration was negative due to the calculation of OD_562_ based on the linear regression of calibration curve. In a previous study on *Magnetospirillum magnetotacticum* MS-1, it was revealed that the fold change in iron concentration between magnetic and nonmagnetic cells is 10 times higher on a dry weight basis.^28^ In our study, the fold change between magnetic (wild type) and nonmagnetic (mutant) MSR-1 cells is approximately 7 times when directly analyzing the iron content in MSR-1 cells. The study of Amor et al.^29^ utilized single-cell inductively coupled plasma mass spectrometry (SC-ICP-MS) to analyze the mass of iron per cell in *Magnetospirillum magneticum* AMB-1. Their results indicated that the mass of iron in AMB-1 ranged from ∼0.1 × 10^–6^ to 2 × 10^–6^ ng per cell when the initial iron concentration in the medium was 100 μM. Referring to the findings of Fernández-Castané, et al.^30^ which established the relationship between OD_565_ and cell concentration in MSR-1, we calculated the mass of iron per cell in our study. The iron concentration obtained from the amended ferrozine assay was divided by the number of MSR-1 cells in a sample. As illustrated in Figure 3, the mass of iron per cell of MSR-1 CCE is 0.7 × 10^–6^ ng per cell, and for MSR-1 magnetic separation sample is 0.6 × 10^–6^ ng per cell. Furthermore, in Figure 4A, the iron mass of magnetosome per cell of MSR-1 with different cell density at OD_565_ ranges from ∼0.4 × 10^–6^ to 0.5 × 10^–6^ ng per cell. Additionally, the mass of iron per cell in B17361 CCE in this study is 1.09 × 10^–7^ ng per cell; the values are consistent with the mutant AMB-1 strain (ΔMAI) in Amor et al.’s study.

Our study was motivated by previous research that employed centrifugation to separate cell debris and PHB or collect magnetosomes from MSR-1 crude cell extracts.^9,10,17,31^ Centrifugation offers the advantage of being a time-saving and convenient method. Our study demonstrated that centrifugation can also be effective in collecting magnetosomes and conducting subsequent analysis. From our results, we observed variations in the total iron content analysis results among MSR-1 cells collected under different cell culture conditions as the centrifugation speed increasing. However, it is important to recognize that while centrifugation provides a means of separating magnetosomes, it may not capture the complete range of magnetosome content but could collect cell debris, which would contribute to the iron concentration. Magnetosomes were found to remain in all the centrifugal-derived supernatant samples (Figure 5D). The inherent magnetic properties of magnetosomes suggest that magnetic separation remains the preferred method for preprocessing, as it offers a more comprehensive analysis that closely reflects the true magnetosome composition. In the study by Rosenfeldt et al.,^9^ the authors performed low-speed centrifugation to remove polyhydroxybutyrate (PHB) from crude extracts of suboxically grown MSR-1. Their results demonstrated that while the PHB content decreased with increasing centrifugation speed in the supernatant, the iron content also decreased due to the high density of magnetosomes. By comparing to sample treated with 0 × *g*, the iron content decreases from approximately 88% to 44% when increasing the centrifugal speed from 250 to 1250 × *g*. In our study, we observed a similar trend (see Figure 5A), with the iron concentration in the supernatant decreasing from 64% to 15% as centrifugation speed increased from 206 × g to 16,708 × *g*. Further treatment of the samples with magnetic separation revealed that magnetosomes were still present in the supernatant after centrifugation at 16,708 × *g* (Figure 5D–F). Moreover, in the study of Rosenfeldt et al.,^9^ the authors analyzed the magnetite crystal size distribution in the supernatant and observed a reduction in the average size of magnetosomes while increasing the centrifugal speed. A previous study has indicated that the size of magnetosomes is influenced by cultural conditions, with microaerobic conditions resulting in smaller magnetosomes compared to anoxic conditions.^32^ In microaerobically derived MSR-1, the presence of a detected iron concentration in the centrifugal-derived samples could be attributed to the small magnetosomes that remain in the supernatant and are difficult to collect. Therefore, although centrifugation can offer some level of effectiveness in magnetosome collection and analysis, magnetic separation remains the recommended approach if a more accurate characterization of the magnetosome content is necessary.

## 4 Conclusion

We repurposed and amended the ferrozine assay for the analysis of iron oxide nanoparticles, incorporating fast, simple, and cost-effective characteristics. The experimental details were thoroughly explored and documented in this study. Upon comparison of the results with those of the known-concentration nanoparticle solution and ICP-OES, the accuracy of the amended ferrozine assay was revealed. However, care should be taken as the iron concentration could be slightly underestimated in the amended ferrozine assay. Next, we further used the amended ferrozine assay in analyzing the crude MSR-1 cell extracts and magnetosomes, which successfully distinguish the magnetosome-containing and -deficient MSR-1 and further reveal the limitation of the MSR-1 cell density in the assay. The amended ferrozine assay also helps us to verify that the suitable method for collecting magnetosomes is magnetic separation.

## Data Availability

All data generated or analyzed during this study are included in this published article and its Supporting Information files.

## Supporting Information Available

Comparison of recovery between the amended ferrozine assay and ICP-OES in different conditions; conditions 1 and 2 indicating Fe_2_O_3_ nanoparticles were digested with 5.0 or 0.4 mL of heating volume and 10 min of heating time; condition 3 indicating standard iron solution was digested with 0.4 mL of heating volume and 10 min of heating time with subsequent supplementation of reductant; all of the experimental conditions were performed in triplicate. Gel electrophoresis of PCR products for amplification of MAI genes from the magnetoaerotaxis-deficient strain B17361, including *mamO* (1899 bp), *mamK* to *mamM* (2014 bp), and *mamQ* to *mamB* (1940 bp); PCR was performed in duplicates; *M* indicates the DNA marker (RTU mass 100 DNA ladder; Protech Technology Enterprise Co., Ltd., Taiwan). Iron concentration of magnetosomes of the variable OD_565_ of MSR-1 that ranged from 0.01 to 10. Iron concentration of magnetosomes from variable OD_565_ of B17361; the OD_565_ concentration of wildtype MSR-1 equals to 2. Iron concentration of MAF^+^ samples; asterisks indicate statistically significant differences in recovery between samples (*: *p* < 0.05; **: *p* < 0.01; ***: *p* < 0.001) (PDF)
