# Supplementary material for: Amended Ferrozine Assay for Quantifying Magnetosome Iron Content in Magnetotactic Bacteria
Source: ACS Omega. 2024 Dec 12;9(51):50650–9. doi: 10.1021/acsomega.4c08607 (PMC11683628; doi:10.1021/acsomega.4c08607)
Supplement: Supplementary file 1 — ao4c08607_si_001.pdf [file ao4c08607_si_001.pdf]

Supplementary Information for

Amended Ferrozine Assay for Quantifying Magnetosome Iron Content in Magnetotactic Bacteria

Ya-Chun Zhao[1], Li-Fen Wu[1], Siang Chen Wu[1,*]

[1] Department of Environmental Engineering, National Chung Hsing University, 145 Xingda Road, Taichung 40227, Taiwan.

*Corresponding author
Siang Chen Wu, Ph.D.
Department of Environmental Engineering, National Chung Hsing University, CEE Building, Room 521,
No.145, Xingda Rd., South Dist., Taichung City, 40227, Taiwan.
E-mail: wusc@nchu.edu.tw;
Tel: +886-4-22840441 ext 521;
Fex: +886-4-22862587

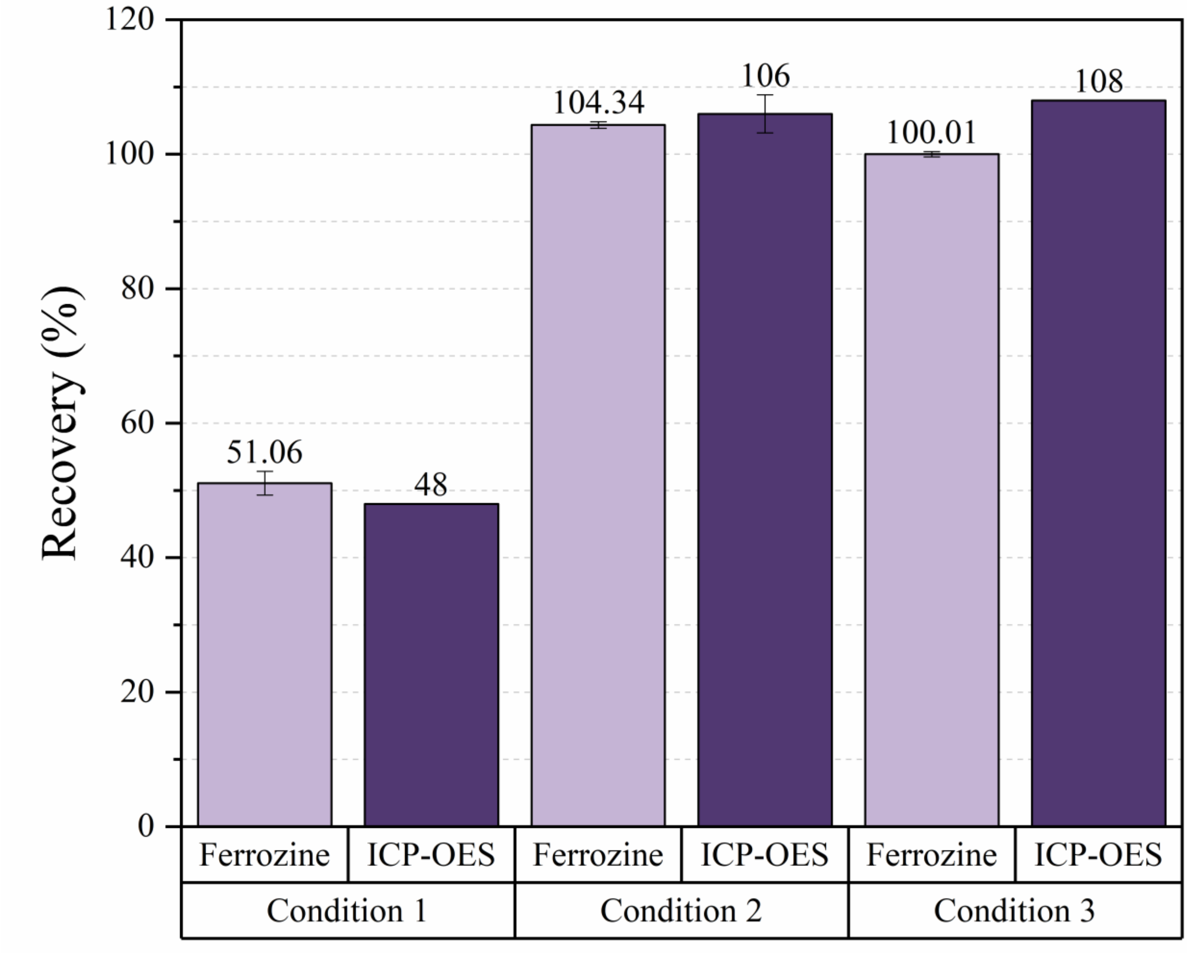

Figure S1 Comparison of recovery between the amended ferrozine assay and ICP-OES in different conditions. Condition 1 and condition 2 indicating Fe_2O_3 nanoparticles were digested with 5.0 ml or 0.4 ml of heating volume and 10 minutes of heating time; Condition 3 indicating standard iron solution was digested with 0.4 ml of heating volume and 10 minutes of heating time with subsequent supplementation of reductant. All of the experimental conditions were performed in triplicate.

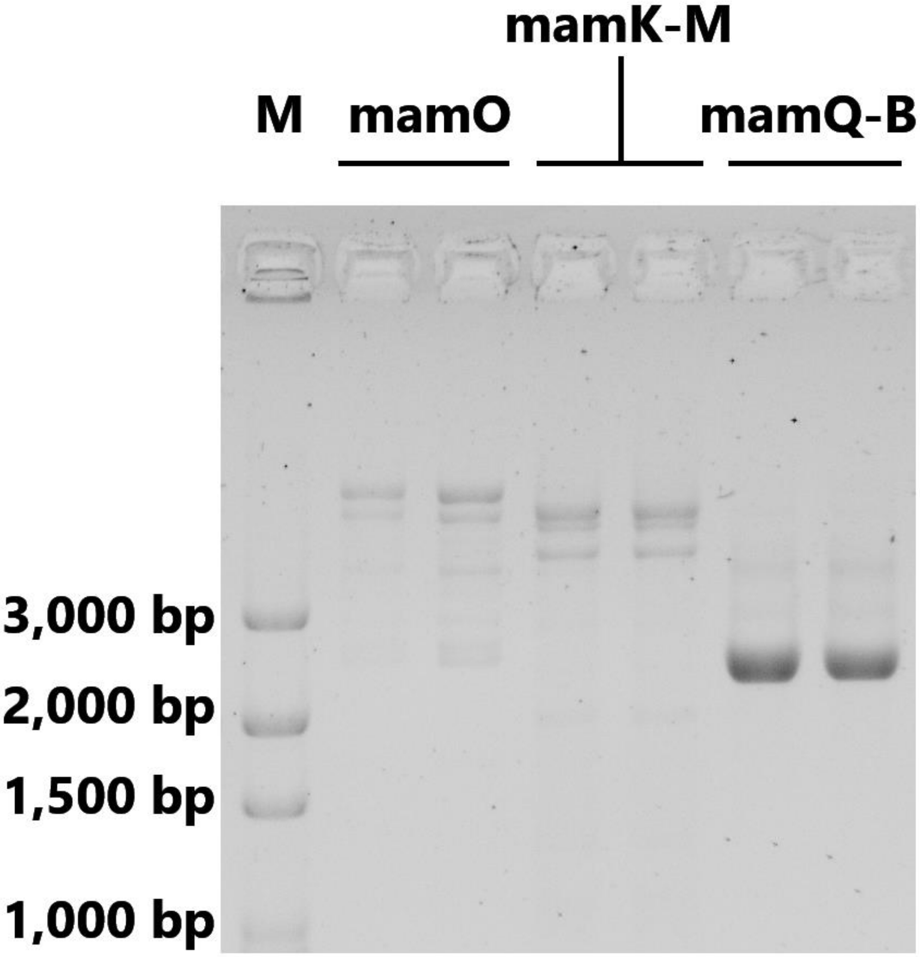

Figure S2 Gel electrophoresis of PCR products for amplification of MAI genes from the magnetoaerotaxis-deficient strain B17361, including *mamO* (1,899 bp), *mamK* to *mamM* (2,014 bp), and *mamQ* to *mamB* (1,940 bp). PCR was performed in duplicates. M indicates the DNA marker (RTU mass 100 DNA ladder; Protech Technology Enterprise Co., Ltd, Taiwan).

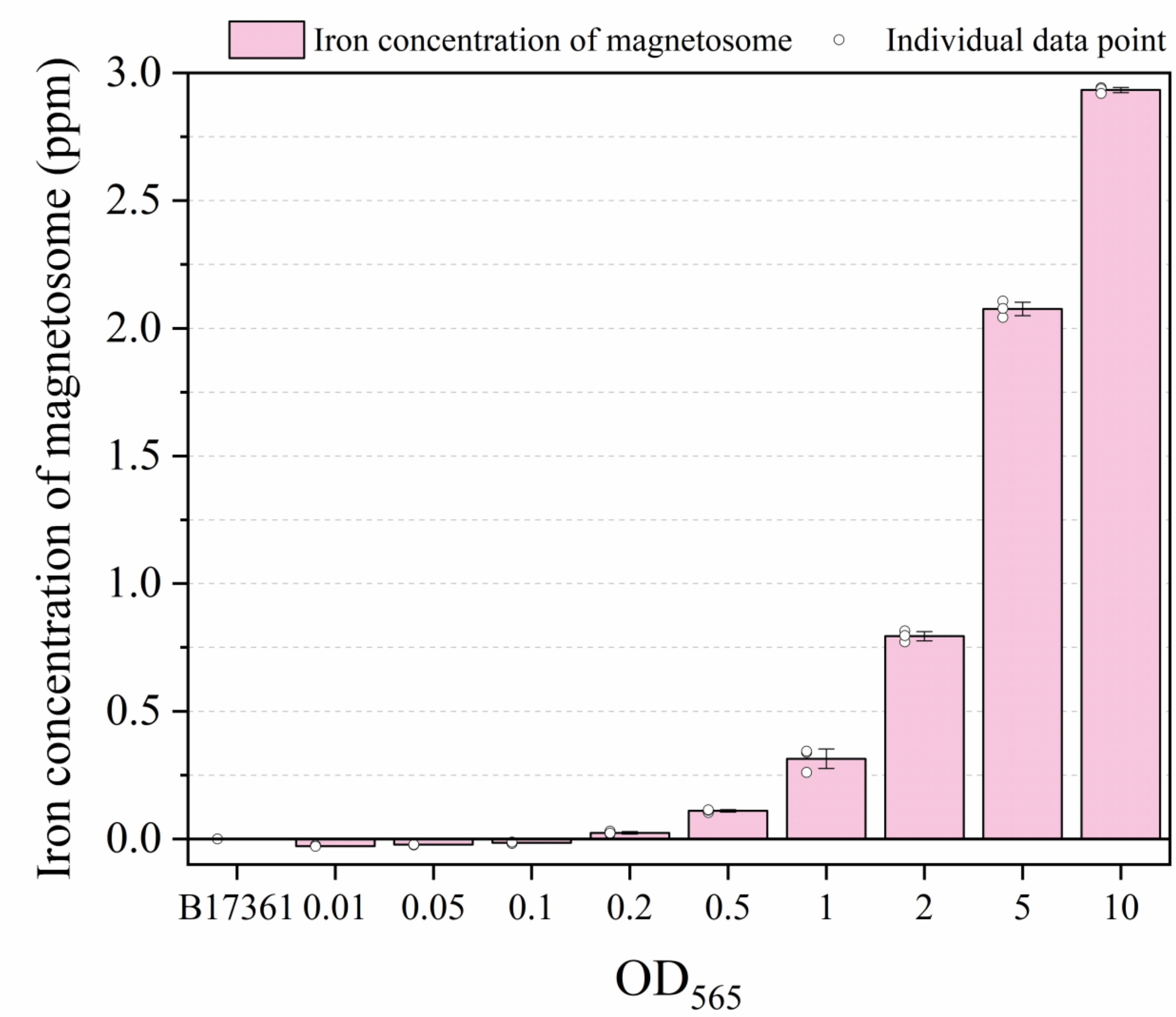

Figure S3 Iron concentration of magnetosomes of the variable OD_{565} of MSR-1 that ranged from 0.01 to 10

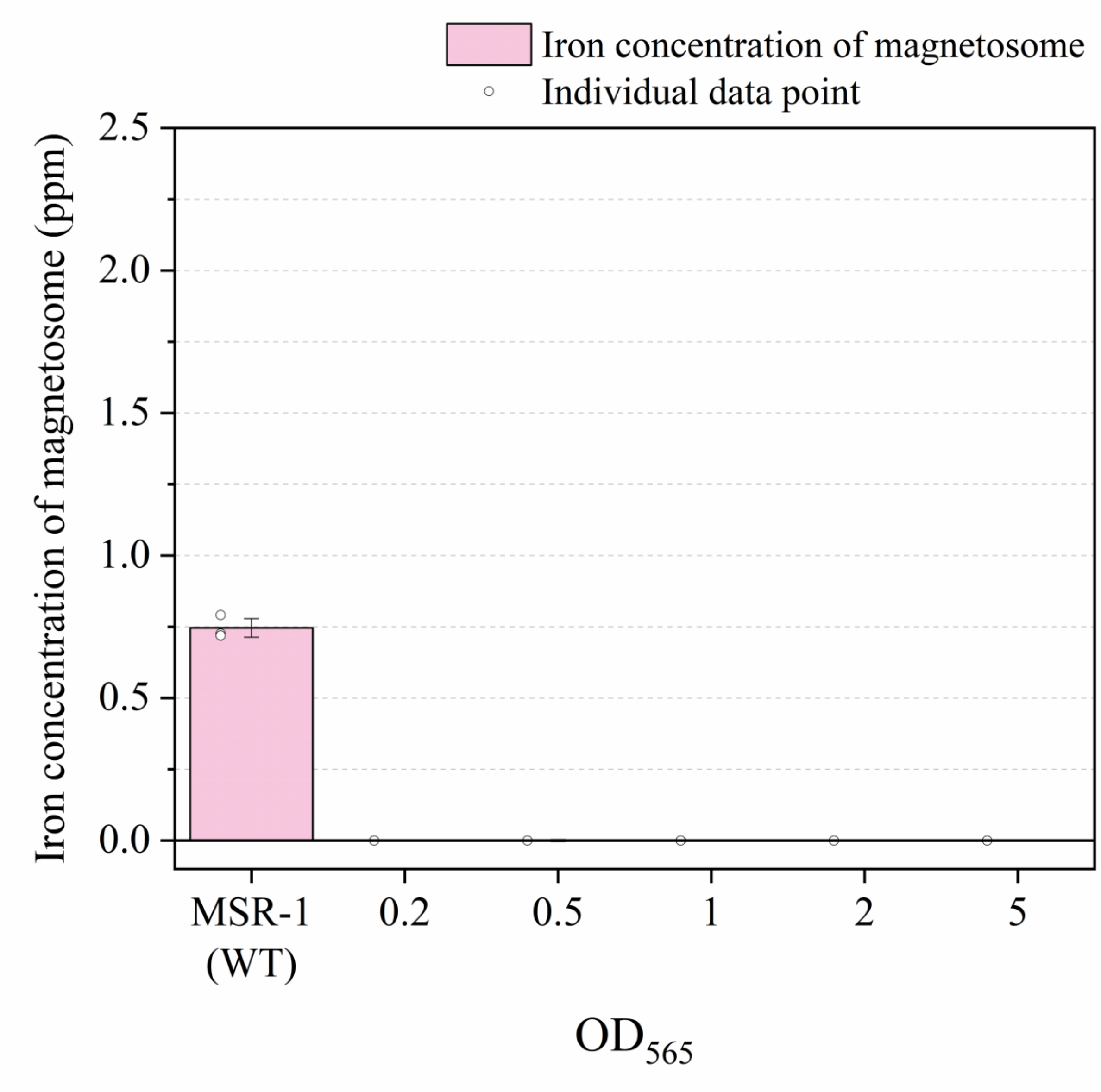

Figure S4 Iron concentration of magnetosomes from variable OD_{565} of B17361. The OD_{565} concentration of wildtype MSR-1 equals to 2.

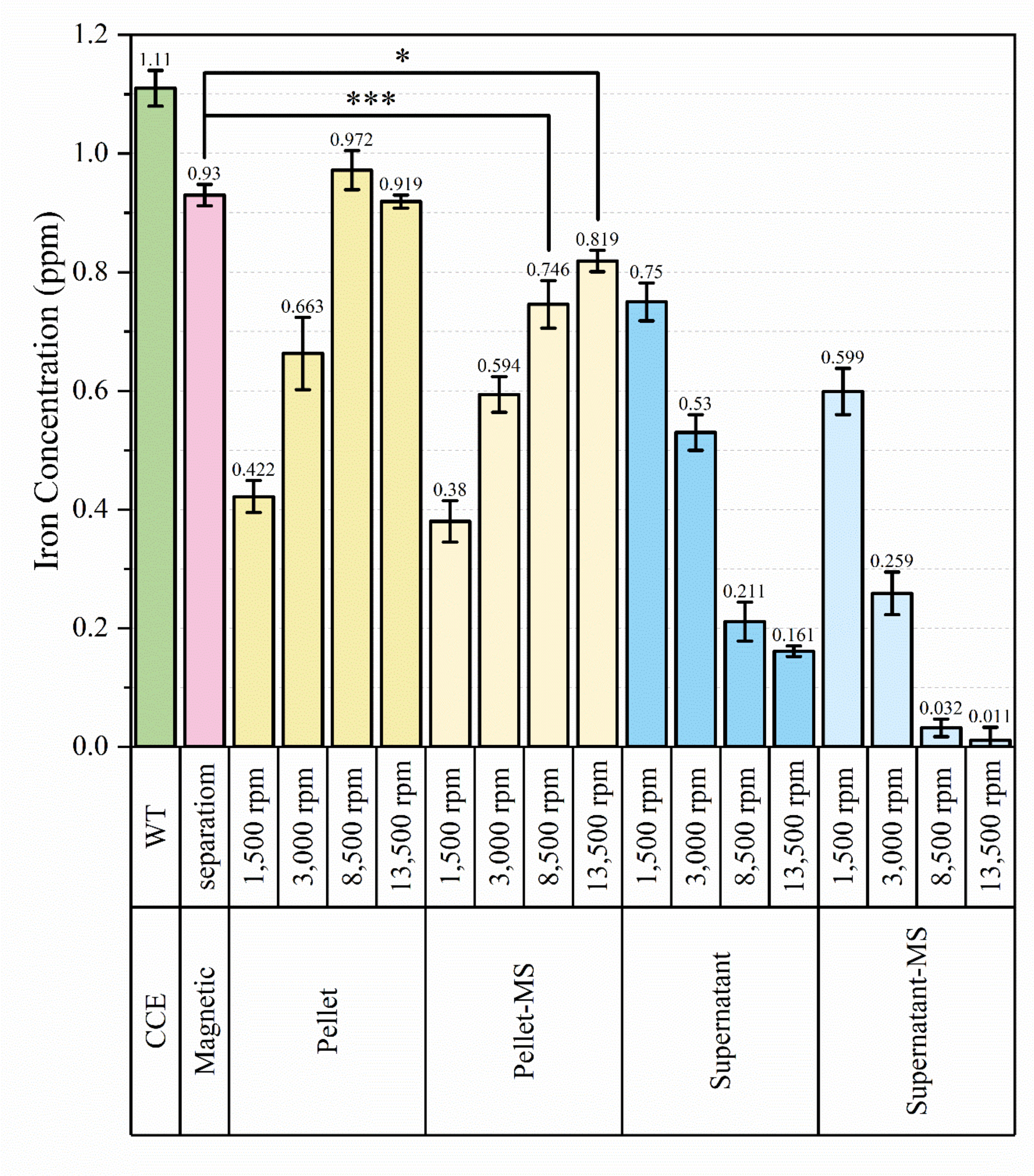

Figure S5 Iron concentration of MAF$^+$ samples. Asterisks indicate statistically significant differences in recovery between samples (*: p<0.05; **: p<0.01; ***: p<0.001).

Table S1 Primer list

Name	Sequence (5'-3')	Reference
mamO_FOR	ATGATTGAAATTGGCGAGACC	This work
mamO_REV	TCACACCGTTGTCAGCATC	
mamK_FOR	GGCTTGAAGCTGCTCGG	
mamM_REV	CTCGGGATCGACGCCAAT	
mamQ_FOR	CGCCAGTTCGGTCGATAAG	
mamB_REV	TCAGACCCGGACCGTC	
